# Still careless: findings from a cross-sectional study of young pedestrians’ risky road crossing behaviors

**DOI:** 10.1186/s13690-020-00421-2

**Published:** 2020-05-18

**Authors:** Mina Hashemiparast, Manoj Sharma, Mohammad Asghari Jafarabadi, Zahra Hosseini

**Affiliations:** 1 Department of Public Health, Maragheh University of Medical Sciences, Maragheh, Iran; 2 Behavioral & Environmental Health, School of Public Health, Jackson State University (Challenging Minds, Changing Lives), 350 West Woodrow Wilson Avenue, Jackson, Mississipi 39213 USA; 3 1.Road Traffic Injury Research Center, Tabriz University of Medical Sciences, Tabriz, Iran; 4 Department of Statistics and Epidemiology, Tabriz University of Medical Sciences, Tabriz, Iran; 5 Social Determinants in Health Promotion Research Center, Hormozgan Health Institute, Hormozgan University of Medical Sciences, Bandar Abbas, Iran

**Keywords:** Pedestrians, Accidents, Injuries, Young adults, Theory of planned behavior

## Abstract

**Background:**

Pedestrian-vehicle collision is one of the most common traffic injuries worldwide. This study aimed to investigate the determinants of pedestrians’ road crossing beliefs and behaviors in potentially risky situations using the Theory of Planned Behavior among Iranian young adults.

**Methods:**

This was a population-based study on a sample of 562 young adults aged 18 to 25 years living in Tehran, Iran. Data were collected by using a self-administered validated questionnaire including constructs of the theory of planned behavior and items of perceived risk and severity. The data were analyzed using independent t-test, analysis of covariance and multivariate analysis of variance.

**Results:**

From all the respondents, 17.8% reported that they had previous experience of vehicle-collision. Among the participants, those who had previous experience of vehicle-collision reported less safety behaviors in crossing the road than those who had not experience an accident. It was found significant differences between participants with and without a history of vehicle-collision for perceived risk (mean difference, adjusted multivariate *P*-value: − 5.77, 0.027) and perceived severity (− 6.08, 0.003), attitude toward traffic regulations (− 6.34, 0.006), attitude toward behavior (− 7.56, 0.005), perceived behavioral control (− 5.20, 0.018), behavioral intention (− 5.35, 0.046) and road crossing behavior in potentially risky situations (− 5.37, 0.004).

**Conclusions:**

Previous unpleasant experience of vehicle-collision is not the only determinant of self-protective behaviors in road- crossing which indicate the role of cognitive and motivational factors such as, subjective norms, attitudes towards risk, feelings of invulnerability in case of facing with vehicle collision.

## Background

Traffic accidents are a rising public health problem worldwide especially in developing countries [1]. In Iran, where about 1.5 million vehicles are added annually, the road traffic injuries and deaths are higher than the global average [2]. Additionally, the fatal traffic collisions in the world are 20 deaths per every 100,000 people while this rate is 33 in Iran [3] and accounts for about 23% of the total disabilities. Further, 17% of the deaths in Iran are attributed to traffic-related accidents [4].

Among the victims of traffic accidents, pedestrians are the most vulnerable road users to traffic injuries. Pedestrians are more at risk than the other road users such as cyclists, users of motorized two-or three- wheelers [5] because in collisions with motor vehicles are not able to protect themselves [6]. Also, it is reported that the fatal pedestrian-vehicle collision is 19 times more than a two-vehicle collision [7].

Moreover, about 45% of fatal traffic injuries in low- and middle-income communities are related to pedestrians, compared to only 18% of traffic fatalities in developed countries. According to the World Health Organization, up to 33% of traffic fatalities in Iran involve pedestrians [8] and among the pedestrians, young adults are an important population that has risky road crossing behaviors [9]. The higher rate of fatalities due to road traffic accidents happen among in the age of 18–25 years in Iran.

Although there are many factors such as environmental, technical, and human factors with pedestrian-vehicle collisions, the human factors more specifically, pedestrians’ risky behavior are tracked in 59% of the vehicle to pedestrian accidents [10, 11]. Hence, identifying and understanding pedestrians’ behaviors in potentially risky situations through the application of social psychological approaches may facilitate developing effective preventive measures.

Among the existing theories, the Theory of Planned Behavior (TPB) is one of the most used models of health-related behaviors [12, 13]. It has been widely studied in the pedestrian context. Such studies have focused more on violations and pedestrian behaviors [14]. For instance a study on the application of the TPB on pedestrians’ self-reported violating crossing behavior intentions indicated that people had a negative attitude toward the behavior of violating road-crossing regulations; they perceived social influences from their family and friends; and they believed that this kind of risky behavior would potentially harm them in a traffic accident [15].

Additionally, it is postulated that pedestrians’ risk perceptions may affect their road crossing behaviors [16]. Numerous fear appeal theories such as protection motivation theory have explained this phenomenon. It is reported that people are more likely to protect themselves if they perceive the risk and severity of a threatening event [17]. As a result, perceiving risky situations may arouse fear and increase awareness of people in identifying potential risks and explaining road-crossing behaviors of pedestrians [18].

Moreover, previous research on pedestrians’ behavior has shown that pedestrian previous history of traffic accidents was one of the determinant predictors of pedestrian’s decision on how to behave while road crossing [19, 20].

One study in Cameroon showed that pedestrians who reported a history of more than three accidents perceived less risk of road crossing and were more adventurous during road crossing [20].

Beyond these studies, little is known about pedestrian behaviors in Iran. Therefore, we aimed to investigate the determinants of pedestrians’ road crossing beliefs and behaviors in potentially risky situations among Iranian young adults using the Theory of Planned Behavior and the variables of perceived risk and severity which come from fear appeals theories.

## Methods

### Study design and participants

A cross-sectional study was conducted to investigate factors affecting pedestrians’ road crossing beliefs and behaviors in potentially risky situations based on the Theory of Planned Behavior.

Since, the young adults (18–25 years old) are the potential victims in pedestrian road traffic accidents in Iran [21] and because of higher rate of mortality due to road traffic accidents among young adults [22], a total of 562 young adults (285 male, 277 female), aged 18–25 years, living in Tehran, Iran were recruited.

A stratified multistage sampling method was applied to recruit participants. After stratifying based on district and size of residence the blocks (units) were randomly selected and every 10th house in a block was selected. Finally, every 18–25 year- old residence from all the 22 districts in Tehran was considered as part of the population of the study. Every household within 22 different districts in Tehran had the same probability of being sampled.

Next, participants responded to the structured written questionnaire of pedestrian road crossing behavior (PEROB) via self-report manner. More detail about the instrument is reported elsewhere [23].

Informed consent was obtained from all study participants before the project began and they had been taught how to complete questionnaires. All of the process of study including data collecting, data analyzing and providing the final report lasted from 27 January 2018 to 20 May 2018.

### Measures

#### TPB theoretical variables

The items which assessed components of the TPB were derived from the developed scale for pedestrian road crossing behavior. There were 18 items for measuring following constructs: (1) subjective norms, (2) attitude, (3) perceived behavioral control, and (4) behavioral intention which all the psychometrics characteristics of this instrument had been reported previously [23].

##### Subjective norms

Four items measured subjective norms toward the risky road crossing behaviors based on two provided scenarios which assessed potentially risky road crossing behaviors. Example of scenarios for measuring subjective norms was “You’ve left your wallet in a bank. When you decide to come back and get it, find yourself on the opposite side of the street that you need to go back and the environmental condition is as follows: 1-The street is a busy two-way arterial and 2-Right side, about 50 meters away from where you are standing, there is a pedestrian bridge, but you are tired and anxious, and you have to get there before closure of the bank. Will you crossing the street without using the pedestrian bridge?” All items were rated on a 5-point Likert scale from 1 to 5 (strongly agree to strongly disagree). The reliability coefficients of (α = 0.79) indicated internal consistency of the measure.

##### Perceived behavioral control

Four other items measured the perceived behavioral control beliefs about pedestrians’ road crossing behaviors (e.g., If I’m in a hurry, I’ll wait in the pedestrian area for the traffic light to turn green). The reliability coefficients of (α = 0.86) verified internal consistency of the measure.

##### Intention

To measure behavioral intention two items (e.g., I intend to keep my road crossing behavior safe in the next 6 months) were used. All items were rated on a 5-point Likert scale from 1 to 5 (strongly agree to strongly disagree). The reliability coefficients of (α = 0.79) indicated internal consistency of the measure.

##### Attitude towards traffic rules and regulations

Attitude was measured using 4 items that negatively worded like ‘traffic rules have been adopted just for drivers’ and ‘walk signals are mainly designated for elderly people and children’ (scored 1 = strongly agree to 5 = strongly disagree). For the current study, the estimates of internal consistency for attitude, as measured by Cronbach’s Alpha, were (α = 0.88).

##### Perceived risk

Perceived risk was measured by presenting a list of risky situations in road crossing (9 items) and asking the respondents to indicate to what extent will involve in an accident when crossing in these situations. These items were categorized on a 5point Likert scale (very small extent scores 5 and very large extent scores 1).

Examples of items were ‘texting messages on the phone while crossing the street’ and ‘crossing the street without using the pedestrian bridge’. An estimated reliability coefficient (α = 0. 83) indicated that the measure of perceived risk was internally consistent.

##### Perceived severity

Perceived severity of involvement in an accident was measured by 3 items including ‘an accident that may happen to me could be costly’, ‘an accident may cause me a permanent disability’, and ‘an accident that may happen to me be able to also troublesome to the driver’. Each item was rated on a 5-point scale (strongly agree scores 5 to strongly disagree scores 1) (α = 0.678). The reliability coefficient for the scale was 0.68. Higher scores on the scale indicated higher perceived severity.

##### Self-reported unsafe road-crossing behaviors

Pedestrian behaviors in potentially risky situations were assessed by 7 items such as: ‘I use the pedestrian “crosswalk” when crossing the street’, and ‘I used to wait until the pedestrian “crosswalk” turns green to cross’. Each item was rated on a five-point Likert scale (never to always). (Positively worded items: never scores 1 and always scores 5; negatively worded items: never scores 5 and always scores 1).

An estimated reliability coefficient (α = 0. 80) indicated that the measure of risky road-crossing behaviors was internally consistent. The total score calculated and transformed into a 0 to 100 result where a higher score represented better condition.

##### Socio-demographics

the socio-demographic variables included age, gender, marital status (single, married) and questions on past involvement in a vehicle-collision (accident history).

### Statistical analyses

Data were analyzed using SPSS statistics for windows version 25.0. The normality of the numeric variables was checked by the Kolmogorov-Smirnov test. Data were presented using mean (SD) for the numeric normal variables and frequency (percentage) for categorical variables. The between-group comparisons of demographic variables were done by the Chi-square test. To determine whether perceived risk and severity, attitude towards traffic regulations and risky road crossing behaviors differed between participants who had and had not been involved in an accident, univariate independent t-test was used and to adjust for sex and education a univariate analysis of covariance (ANCOVA) was utilized.

Additionally, to compare all constructs of TPB between participants with and without history of vehicle-collision, a Multivariate Hoteling T2 Tests based on Wilks’ Lambda was conducted and also to adjust for sex and literacy level, multivariate ANCOVA based on Wilks’ Lambda was done. *P*-values less than 0.05 were considered as significant.

## Results

In all 562 young pedestrians were studied (285 males, 277 females). The mean (SD) age of participants was 22.08 (2.35) years. One hundred (17.8%) of participants had previous experience of vehicle collisions. The detailed socio-demographic characteristics of those with and without a history of vehicle collision are shown in Table 1.There was a significant difference in gender distribution between those with and without a history of vehicle collision (*p* < 0.05), but for other variables this difference was not significant.

**Table 1:** Socio-demographic characteristics of the participants with and without previous experience of vehicle-collision from a cross-sectional study of young pedestrians’ risky road crossing behaviors, Tehran, Iran (2018)

	With a history of vehicle-collision (***n*** = 100) n (%)	Without a history of vehicle-collision (***n*** = 462) n (%)	***P***-Value
**Age (Year)**			
18	4 (4.0)	23 (5.0)	0.769
19	13 (13.0)	64 (13.9)	
20	15 (15.0)	58 (12.6)	
21	10 (10.0)	58 (12.6)	
22	8 (8.0)	52 (11.3)	
23	11 (11.0)	49 (10.6)	
24	22 (22.0)	68 (14.7)	
25	17 (17.0)	90 (19.5)	
**Gender**			
Male	63 (63.0)	214 (46.3)	0.002
Female	37 (37.0)	248 (53.7)	
**Marital Status**			
Single	87 (87.0)	401 (87.0)	1.000
Married	13 (13.0)	60 (13.0)	
**Education**			
Primary	0 (0.0)	3 (0.6)	0.155
Secondary	41 (41.0)	145 (31.4)	
Higher	59 (59.0)	314 (68.0)	
**Employment**			
Student	49 (49.0)	244 (53.9)	0.138
Employed	30 (30.0)	123 (27.2)	
Housekeeper	2 (2.0)	30 (6.6)	
Unemployed	19 (19.0)	56 (12.3)	

# *P*-Value based on exact Chi-square test

Univariate analysis showed significant differences between participants with and without a history of vehicle-collision for perceived risk and severity, attitude toward traffic regulations, attitude toward behavior, subjective norms, perceived behavioral control, behavioral intention and road crossing behavior in potentially risky situations, each individually. The same results were observed after adjusting for sex and education level of the participants. Moreover, according to multivariate analysis, significant differences were observed between participants with and without a history of vehicle-collision for all variables simultaneously. The same results were observed after adjusting for sex and education level of participants.

The results of the univariate and multivariate tests comparing the constructs between the two groups are shown in Table 2.

**Table 2:** Univariate and multivariate tests comparing the theory of planned behavior (TPB) constructs between participants with (n = 100) and without (n = 462) previous experience of vehicle-collision from a cross-sectional study of young pedestrians’ risky road crossing behaviors, Tehran, Iran (2018)

				Unadjusted		Adjusted	
Variables	Experience of vehicle-collision	Mean (SD)	Mean Difference (95% CI)	Univariate ***P***-value ^$^	Multivariate ***P***-value ^#^	Univariate ***P***-value ^$$^	Multivariate ***P***-value ^##^
**Perceived risk**	Yes	48.18 (23.35)	−5.76 (−10.92 to −0.61)	0.028	0.007	0.027	0.039
	No	53.94 (23.87)					
**Perceived severity**	Yes	80.10 (19.51)	−6.08 (−9.50 to −2.66)	0.001		0.003	
	No	86.18 (14.85)					
**Perceived behavioral control**	Yes	62.71 (17.29)	−5.20 (−8.88 to −1.53)	0.006		0.018	
	No	67.91 (16.87)					
**Subjective norms**	Yes	60.98 (23.07)	−4.53 (−9.21 to 0.15)	0.058		0.89	
	No	65.51 (21.27)					
**Behavioral intention**	Yes	57.46 (20.43)	−5.35 (−9.98 to −0.71)	0.024		0.046	
	No	62.81 (21.60)					
**Attitude toward behavior**	Yes	59.66 (23.50)	−7.56 (−12.35 to −2.77)	0.002		0.005	
	No	67.22 (21.81)					
**Attitude toward traffic regulations**	Yes	66.95 (16.28)	−6.34 (−9.94 to −2.73)	0.001		0.006	
	No	73.29 (16.72)					
**Road crossing behavior in potential risky situations**	Yes	65.19 (16.61)	−5.37 (−8.62 to −2.12)	0.001		0.004	
	No	70.56 (14.65)					

#: Multivariate Hoteling T2 Tests based on Wilks’ Lambda

##: Multivariate Analysis of Covariance (ANCOVA) based on Wilks’ Lambda adjusting for sex and education

$: Univariate Independent t-test

$$: Univariate ANCOVA adjusting for sex and education

Higher score indicates better condition

## Discussion

The study found that young pedestrians who had previous experience of vehicle-collision were still careless and repeated their risky behaviors while crossing the street. These findings are in line with the scientific literature that is summarized in the following quote: “personal experience is a complex determinant of self-protective behaviors” [24].

The present study results suggest that in addition to previous unpleasant experiences, other attitudinal and motivational factors also contribute to the pedestrians’ decision making while crossing the street in potentially risky situations.

The results revealed the role of perceived risk as the attitudinal factor that predicts road-crossing behavior. We found that pedestrians who reported having been involved in a vehicle-collision perceived risk of unsafe road-crossing as less risky compared with those who had not, and reported more risky behaviors in road-crossing.

This finding is consistent with health behavior theories such as the Health Belief Model and Protection Motivation Theory. According to these theories, the low perceived risk leads to higher risk-taking behavior [25]. Hence, we would expect that persons who decide to cross the street in potentially risky situations are more likely to perceive this behavior as positive and easy to do; they are, therefore, more exposed to dangerous decisions. This may indicate that this is because they had become unrealistically optimistic when judging their injury risk. This interpretation is consistent with research in risky driving showing that drivers are illusory optimistic when judging their driving competency and traffic-crash risk [26].

According to the decision-making perspective that explains the relationship between perception of risk and the adoption of protective behaviors, if individuals perceive their vulnerability to the risk take protective behaviors [20]. A study in China about individuals’ intention to drive after drinking showed that feeling of invulnerability influenced intention by promoting favorable attitudes toward driving after alcohol use [27].

Moreover, the present study showed perceived severity was a significant predictor of pedestrians’ unsafe road-crossing behaviors. Accordingly, fewer perceived severity of involvement in an accident was associated with higher risky road crossing behaviors. This finding suggests that perceived severity is influenced by previous experience to violate traffic laws and a belief that, if one has had previous non-fatal traffic injuries, he is likely to repeat risky road-crossing behaviors. This interpretation is consistent with research showing that successful past experience to violate traffic rules would potentially prompt the probability of repeating offenses [28].

The present study also examined the role of attitudes towards traffic rules and regulations. According to findings, pedestrians with previous experience of vehicle-collision indicated more negative attitudes towards traffic rules and regulations than individuals without such experience. These groups were also more likely crossing the street in potentially risky situations than the other group. According to the theory of planned behavior, attitudes predict future behaviors [15].

In a study of pedestrian intention to violate traffic regulations, Diaz found that people who had a positive attitude towards illegal road-crossing reported more violations and errors as pedestrians [29].

This finding also is in line with previous studies on pedestrian behaviors showing that pedestrians with negative attitudes toward traffic safety facilities, show more risky pedestrian behaviors [30]. In general, the findings support the view that attitudes towards traffic rules and regulations predict the involvement in risky road-crossing behaviors.

In examining the constructs of the TPB, there are significant differences in some components of the TPB between individuals with and without a history of vehicle-collision. Based on the results, individuals who had experience of vehicle-collision had low perceived behavioral control and thought it would easy to cross the street in potentially risky situations. A possible explanation is that those persons who consider it easy to cross the street in potentially risky situations have less perceived risk. This interpretation may be inconsistent with Evans & Norman’s study which found that there was a link between perceptions of control and perceived risk in as much as those road crossing behaviors which are seen to be easy to perform may be associated with low perceptions of risk [15].

It is postulated that attitude could act as antecedent of behavior [31, 32]. Consequently, pedestrians’ attitude toward road crossing behavior would be determinant factor for individuals to protect her/him alongside of environmental modifications. It seems, in the context like Iran, educating of road crossing behavior for schoolchild students in the schools would be effective to improve student’s attitude toward behaving safe in later life and could improve communities’ value toward safe citizenship. Finally, although, it was not found the total rate of pedestrians’ accident rate among population of Iran, our findings, indicated that 17.8% of the participants had prior history of accidents as pedestrians. Based on the reported evidences in Iran, pedestrians account for more than 30% of accidents and 23% of road deaths among all reported road traffic accidents [33], where, the traffic accidents in Iran with an annual incidence rate of 26.5 per 100,000 populations, are the second leading cause of death and also, the Iran Legal Medicine Organization has reported that pedestrians account for 25% of the total road traffic deaths in 2013 in Iran [34]. As a result, this condition of pedestrians’ crash patterns has become one of the main concerns of traffic safety professionals and how to overcome on this problem.

On the other hand, there are evidences that indicate previous involving in non-fatal crashes develop posttraumatic stress disorders (PTSD). PTSD affects pedestrians and passengers as well as drivers [35]. Hence, inconsistent with our main result, some of individuals who have previous experience of motor vehicle accidents perceived more threat and showed more cautious in road crossing behaviors [36]. Further research is needed to investigate the effects of personal experience on attitude and self-protective behaviors.

There were some limitations to the study. The actual behaviors of participants were not observed in the real condition on the roads. Other studies used this approach [28, 29]. Next, it used self-reported measures, which might have resulted in inaccurate recall and/or reporting. To minimize bias to some extent, we used inverted scoring for some items. Although the TPB has inspired a considerable amount of empirical health behavior research but due to the cross-sectional nature of the study, no causal inferences could be drawn and consequently limited our explanation for predictability of TPB for the vehicle collision. In this regard, ideally a cohort study would be required. Moreover the study’s design cannot assess whether significant differences in psychological traits and attitudes between those with and without a history of pedestrian-vehicle collision have changed in magnitude before and after the accidents.

## Conclusion

Our findings suggest that previous unpleasant experience of vehicle-collision is not the only determinant of self-protective behaviors in road- crossing. In other words, previous experience of vehicle-collision doesn’t prohibit youth people to be careful about current behaviors, which make this question that why in spite of experience of vehicle-collision, young pedestrians still are inattentive about themselves. This study also highlights that emphasizing on personal commitment to protect him/her should be supported and associated by improving the community value to obey policies and norms which provide safety roads for pedestrians.

## Data Availability

The datasets used and analysed during the current study are available from the corresponding author on reasonable request.

## Abbreviations

TPB: Theory of Planned Behavior
PEROB: Pedestrian Road Crossing Behavior
ANCOVA: Analysis of Covariance
SD: Standard Deviation
